# Seroprevalence of measles, mumps, rubella, varicella–zoster and hepatitis A–C in Emirati medical students

**DOI:** 10.1186/1471-2458-12-1047

**Published:** 2012-12-05

**Authors:** Mohamud Sheek-Hussein, Rayhan Hashmey, Ahmed R Alsuwaidi, Fatima Al Maskari, Leena Amiri, Abdul-Kader Souid

**Affiliations:** 1 Department of Community Medicine, UAE University, College of Medicine and Health Sciences, P.O. Box 17666, Al-Ain, UAE; 2 Department of Infectious Diseases, Tawam Hospital, Al Ain, UAE; 3 Department of Pediatrics, United Arab Emirates University, Al Ain, UAE; 4 Department of Psychiatry, United Arab Emirates University, Al Ain, UAE

**Keywords:** Medical student, Immunization, Blood borne, Transmission, Prevention, UAE

## Abstract

**Background:**

The aims of this study were to assess the seroprevalence of vaccine-preventable infections in Emirati medical students, and to provide scientific evidence for implementation of a cost-effective immunization guideline and policy for medical school admission.

**Methods:**

This prospective cohort study involved 261 (61% female) Emirati medical students (preclinical and clinical) attending the College of Medicine and Health Sciences at UAE University. Data on vaccination and history of infectious diseases were collected from participants. Blood samples were collected between July 1, 2011 and May 30, 2012 for serological testing and QuantiFERON®-TB assay.

**Results:**

All students tested negative for infection with hepatitis C virus and human immunodeficiency virus. The prevalence of seropositivity to rubella virus was 97%, varicella–zoster virus 88%, mumps virus 84%, measles virus 54%, hepatitis B virus (HBV) 48%, and hepatitis A virus 21%. The QuantiFERON®-TB test was positive in 8% and indeterminate in 2%. Forty percent of students received HBV vaccine at birth; their HBV titers (mean ± SD) were 17.2 ± 62.9 mIU/mL (median = 1.64). The remaining 60% received it at school and their titers were 293.4 ± 371.0 mIU/mL (median = 107.7, *p* = 0.000).

**Conclusion:**

About 50% of students were susceptible to HBV and measles virus; therefore, pre-matriculation screening for antibodies against these viruses is highly recommended. Moreover, tuberculosis screening is necessary because of the high influx of expatriates from endemic areas. Students with inadequate protection should be reimmunized prior to contact with patients.

## Background

Immunity against vaccine-preventable and other transmissible infections is of major importance, especially in medical students who are at increased risk of exposure to infectious body fluids [1]. Thus, pre-matriculation screening and immunization policies are much needed for medical school admission. Implementation of cost-effective programs, however, requires surveillance that identifies immunization gaps and susceptibility to infectious diseases.

In one study, about 50% of medical students reported exposure to infectious body fluids and 0.3% reported a needle stick injury [2]. Even higher incidences of exposure to infectious body fluids were found in other studies [3,4]. One case report describes a student who contracted encephalitis associated with measles [5]. A recent report from Australia pointed out that many medical students were not immune to vaccine-preventable infections, and their self-reported history of previous vaccination was often inaccurate [6].

The United Arab Emirates (UAE) was the first among the Gulf Council Countries to adopt the WHO Expanded Program on Immunization (EPI). As a result, childhood vaccine-preventable diseases have almost been eliminated, with an immunization coverage reaching 98% [7]. However, immunity against some of these infections weans off in time and results in unsustainable protection in adults [8].

The aims of this study were to assess serological immunity against vaccine-preventable and other transmissible infections in Emirati medical students, and to provide scientific evidence for development of effective pre-matriculation screening and immunization policies for our students during the admission process.

## Methods

### Study participants and data collection

This prospective study involved a cohort of Emirati medical students attending the College of Medicine and Health Sciences at UAE University. Among 397 matriculated students, 319 (80%) consented and enrolled in the study. Between July 1, 2011 and May 30, 2012, blood was collected from 261 (82%) students [158 (61%) female]. The remaining 58 (18%) students had time conflicts with classes and delayed the testing. The only exclusion criterion was not consenting to the study. Details about age were only available for 177 (68%) students: mean ± SD, 21.2 ± 2.3 years of age; median, 21 years; range, 16–33 years of age.

Blood was collected from all 261 students for measuring antibody titers against measles, mumps and rubella viruses, varicella–zoster virus (VZV), hepatitis A virus (HAV), hepatitis B virus (HBV), and hepatitis C virus (HCV). Moreover, they were all requested to undergo human immunodeficiency virus (HIV) antigen and antibody and QuantiFERON®-TB Gold tests. Missing test values were caused by various factors, including inadequate serum volume to run all the tests, and a shortage of reagents or analytical kits.

A brief self-administered questionnaire (provided by 319 students) was used to collect demographic data, which included immunization and medical histories. Students were also asked to bring their official immunization records (provided by 91 students).

The study was approved by the institutional review board for protection of human subjects (Assessment of Immunization Status of Vaccine-Preventable Diseases for Cohort Medical Students at College of Medicine and Health Sciences, UAE University; reference number 10/59). Informed consent was obtained from each participant.

### Laboratory testing

Blood was collected for measuring antibody titers against measles virus (VIDAS® Measles IgG, REF 30 219; Biomerieux, Lyon, France); mumps virus (VIDAS® Mumps IgG, REF 30 218; Biomerieux); rubella virus (Rubella IgG, REF 6C17, 840627/R3; Abbott, Wiesbaden, Germany); VZV (VIDAS® Varicella–Zoster IgG, REF 30 217; Biomerieux); HAV (HAVAb-IgG, REF 6C29, 36-6800/R1, B6C290; Abbott); HBV (Anti-HBs, REF 7C18, 48-8436/R7, B7C180; Abbott); and HCV (Anti-HCV, REF 6C37, 48-6176/R6, B6C370; Abbott). HIV antigen and antibody tests (HIV Ag/Ab Combo, REF 4 J27, 49-9527/R01, B4J2S0; Abbott) and QuantiFERON®-TB Gold (In-Tube Method; Cellestis, Hannover, Germany) were also performed. All tests were performed and all results were interpreted according to manufacturers’ instructions.

### Statistical analysis

The data were summarized by arithmetic mean and standard deviation. Mann–Whitney *U* test was used for nonparametric values; *p* < 0.05 was statistically significant.

## Results

**Figure 1:**
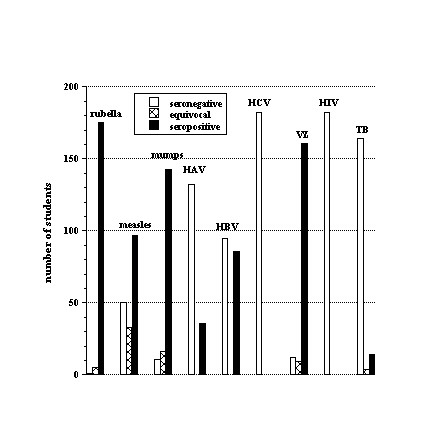
Seroprevalence of rubella, measles, mumps, HAV, HBV, HCV, VZV, HIV and QuantiFERON®-TB in the Emirati students.

All tested students were nonreactive for HCV and HIV. VZV serology was available in 182 (70%) of 261 students. One hundred and sixty-one (88%) students were positive, nine (5%) were equivocal, and 12 (7%) were negative (Table 1 and Figure 1).

**Table 1:** Seroprevalence and result of QuantiFERON®-TB assay of the studied transmissible infections in Emirati medical students

**Results**	**Rubella**^**1**^	**Measles**^**2**^	**Mumps**^**3**^	**HAV**^**4**^	**HBV**^**5**^	**HCV**^**4**^	**VZ**^**6**^	**HIV**^**7**^	**QuantiFERON®-TB**^**7**^
Positive (%)	96.5	54	84	21	48	0	88	0	8
Equivocal (%)	3	18	10	-	-	-	5	-	2
Negative (%)	0.5	28	6	79	52	100	7	100	90

^1^A positive result is >10 IU/mL, equivocal 5.0 to 9.9 IU/mL, and negative 0.0 to 4.9 IU/mL. Results were available in 181 (69%) of 261 students.

^2^A positive result is >0.70 relative fluorescence value (RFV), equivocal >0.50 to <0.70 RFV, and negative <0.50 RFV. Results were available in 180 (69%) of 261 students.

^3^A positive result is >0.50 RFV, equivocal >0.35 to <0.50 RFV, and negative <0.35 RFV. Mumps serology was available in 170 (65%) of 261 students.

^4^A reactive test is >1.0 relative light unit (RLU) and nonreactive <1.0 RLU. Results were available in 168 (64%) of 261 students.

^5^A reactive test is >10.0 mIU/mL and nonreactive <10.0 mIU/mL. Results were available for 181(69%) of 261 students.

^6^A positive result is >0.90 RFV, equivocal 0.60 to <0.90 RFV, and negative <0.60 RFV. The titer was available in 182 (70%) of 261 students.

^7^Please refer to the manufactures’ brochures. Results were available only in 182 (70%) of 261 students. Equivocal test indicates indeterminate.

Positive tests for HAV, HBV, HCV and HIV indicate reactive and negative tests indicate non-reactive.

**Figure 2:**
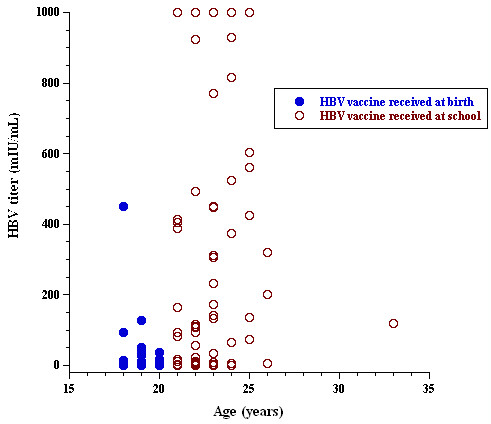
Seroprevalence of HBV titer as a function of student age.

HBV serology was available in 181 (69%) of 261 students; 95 (52%) had a titer <10.0 mIU/mL (nonreactive) and 86 (48%) had a titer >10.0 mIU/mL (reactive) (Table 1 and Figure 1). Of 136 students with a matched age and HBV serology, 55 (40%) were <20 years of age and received vaccination at birth, and had mean HBV titers of 17.2 ± 62.9 mIU/mL (median = 1.64). The remaining 81 (60%) students were >20 years of age and received vaccination at school, and had mean HBV titers of 293.4 ± 371.0 mIU/mL (median = 107.7, *p* = 0.000). Of the 55 students who received HBV vaccine at birth, 44 (80%) were nonreactive. By contrast, of the 81 students who received HBV vaccine at school, only 27 (33%) were nonreactive (*p* = 0.000). Fifty-six students presented official records of HBV vaccination at birth and had HBV titers of 62.1 ± 195.4 mIU/mL (median = 1.8); 39 (70%) students were nonreactive and 17 (30%) were reactive. By contrast, 17 students presented official records of HBV vaccination at school and had HBV titers of 216.8 ± 263.7 mIU/mL (median = 4.2, *p* = 0.000); nine (53%) students were nonreactive and eight (47%) were reactive (Figure 2).

HAV serology was available in 168 (64%) of 261 students. Thirty-six (21%) students were reactive and 132 (79%) were nonreactive (Table 1 and Figure 1).

Rubella serology was available in 181 (69%) of 261 students. The cutoff for positive rubella titer was 10 IU/mL and equivocal titer >5 and <10 (Table 1 and Figure 1). Of the 181 students, 175 (96.5%) had a rubella titer of >11 IU/mL (reactive); one (0.5%; 18-year-old female student) had a titer of 3 IU/mL (nonreactive) and five (3%; three female and two male) had a titer of 9 IU/mL (equivocal). Thus, all students had some immunity to rubella, except for one female student.

**Figure 3:**
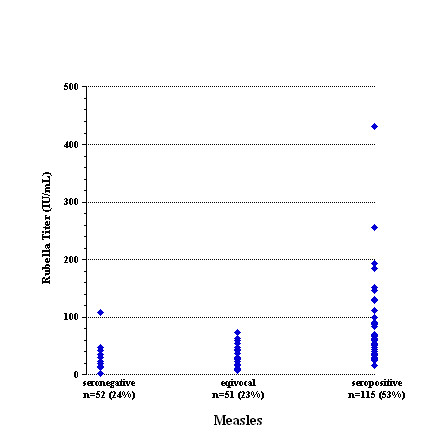
Seroprevalence of measles as a function of rubella titer.

Measles serology was available in 180 (69%) of 261 students. Fifty (28%) students were negative/nonreactive, 33 (18%) were equivocal, and 97 (54%) were positive/reactive (Table 1 and Figure 1). Student age did not significantly influence measles seroprevalence; of 136 students with a matched age and measles serology, 39 (29%) were seronegative, 18 (13%) were equivocal, and 78 (57%) were reactive. By contrast, rubella titers for students with nonreactive (or equivocal) measles were 41 ± 40 IU/mL (n = 103) and with reactive measles 66 ± 57 IU/mL (n = 105; *p* = 0.000) (Figure 3).

**Figure 4:**
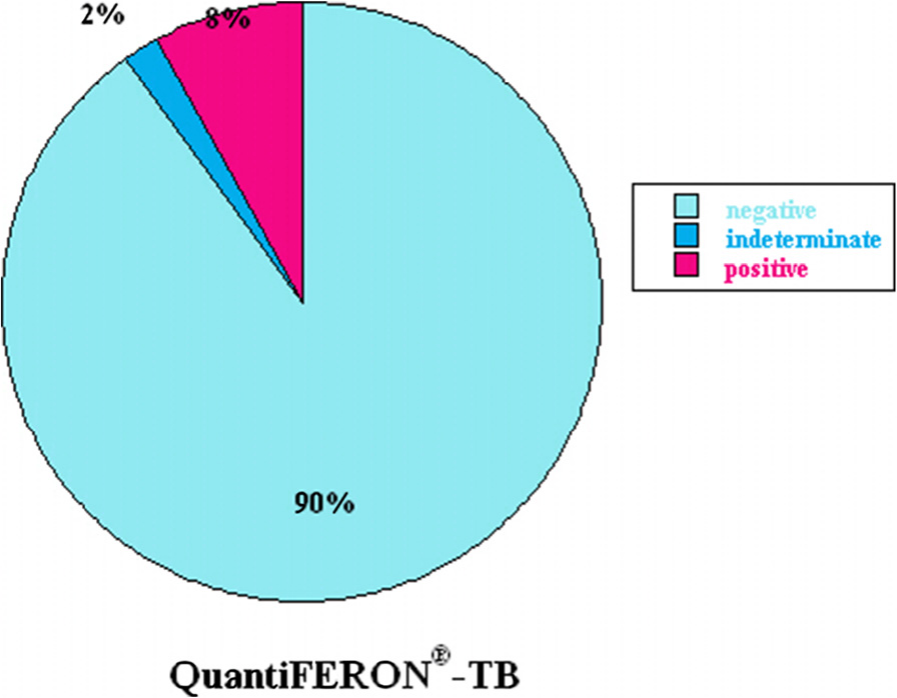
Summary of the results of QuantiFERON®-TB Gold test for tuberculosis.

Mumps serology was available in 170 (65%) of 261 students. Eleven (6%) students were nonreactive, 16 (10%) were equivocal, and 143 (84%) were reactive (Table 1 and Figure 1). The QuantiFERON®-TB test was available in only 182 (70%) of 261 students. Fourteen (8%) students were reactive, four (2%) were indeterminate, and 164 (90%) were nonreactive (Figure 4). The age range of abnormal QuantiFERON®-TB test was 19–25 years, which did not significantly differ from those with a negative test (Table 1 and Figure 1).

## Discussion

Although all students reported receiving childhood immunization, the data here show that approximately 50% of the students tested were susceptible to HBV and approximately 46% to measles (equivocal plus negative). These results are consistent with recent reports from Germany and Switzerland [1,8]. The students were offered reimmunization for these diseases. Significantly, 8% of students had positive and 2% had indeterminate QuantiFERON®-TB Gold test for tuberculosis (TB). After excluding active TB disease, these students were offered treatment of latent disease with a standard regimen of isoniazid and vitamin B6 for 9 months.

Medical students are often exposed to infectious materials during their clinical rotations [1-3,9]. Therefore, lack of immunity to vaccine-preventable diseases may predispose them, their patients and healthcare workers to serious sequelae. Thus, it is important to identify susceptible students and implement effective reimmunization policy during school admission. The findings here show significant immunization gaps in medical students, especially for HBV and measles virus. Rubella and mumps viruses and VZV, on the other hand, are reasonably covered.

According to a report in 1994 from the university of Sydney, 22% of medical students had one or more needle stick injuries in the previous 2 years [9]. In another survey, 14–30% of medical students reported a needle stick injury [3]. Consequently, risk of exposure to HBV, HCV and HIV is substantial [4]. In our study, 18 of the 261 (6.8%) students reported needle stick injury.

The annual incidence of acute hepatitis in the United Kingdom between 1985 and 1988 was 4 per 100,000 in adult males and 2 per 100,000 in adult females, and it was estimated to be 25 per 100,000 surgeons [10]. More importantly, studies have shown protective efficacy (~90%) of HBV and other vaccines in healthcare workers [11,12].

UAE has intermediate endemicity with hepatitis B surface antigen (HBsAg) carrier rates of 2–5% [13]. HBV vaccine was introduced in the UAE in October 1991; all infants received three doses of vaccine (at 0, 2 and 6 months of age). Moreover, all school students born before October 1991 were vaccinated with three doses of vaccine by the year 2000 (Annual Reports, 1992 and 2002; Preventive Medicine Department, Ministry of Health, UAE). Therefore, it is expected that Emirati students <20 years of age would have received the first dose of vaccine at birth, while those aged >20 years would have been expected to receive it at school. It has been reported that among immunocompetent children who respond to the complete primary three-dose vaccination series with anti-HBsAg concentrations of >10 mIU/mL, 15–50% have low or undetectable concentrations of anti-HBsAg at 5–15 years after vaccination [14]. Adults were noted to have rapidly decreasing anti-HBsAg titers within the first year after primary vaccination; titers of <10 mIU/mL were observed in 30–60% within 9–11 years after vaccination [14]. This finding is consistent with the titers of <10 mIU/mL found in 52% of the studied students (Table 1 and Figure 1). Several studies have demonstrated excellent immune memory (anamnestic response to vaccine challenge) in those individuals and that acquisition of HBsAg is rare despite low or undetectable antibody levels several years after vaccination [15]. Therefore, a booster dose may not be necessary in fully vaccinated healthy people. The notion is not applicable to immunocompromised subjects, such as those with HIV infection and renal failure [15].

Data on the seroprevalence of HAV in UAE are lacking. The only available information comes from 1995–1996, when 960 samples were examined and showed that 60% of the population had antibodies against HAV by the age 17–20 years (intermediate endemicity) [16]. Only 21% of the studied students had anti-HAV antibodies (Table 1 and Figure 1). This decline in the seroprevalence of HAV probably reflects the changing socioeconomic conditions.

VZV vaccine has been available in the UAE for >10 years, but it has just recently been introduced to the national immunization program [17]. VZV is highly contagious and immunity is often acquired from natural infection. This explains the relatively high seropositive rate among the studied students. The IgG seronegativity (or equivocal) rate to VZV was 12% (Table 1 and Figure 1). In 2001, Uduman et al. reported that varicella seropositive rates among Emirati citizens increased with age: <10 years, 45.8%; 11–20 years, 68.4%; 21–30 years, 89.5%; 31–40 years, 94.7%; and >41 years, 88.9% [18]. Varicella infection in adults may lead to serious complications. A study on varicella infection among hospitalized adults in UAE reported a mortality rate of 4.9% and a high rate of complications, such as pneumonia (28.4%), skin infection (25.4%), septicemia (10.7%), encephalitis/meningitis (8.8%), acute respiratory distress syndrome (6.8%), renal failure (2.9%), and hepatic failure (1.9%) [19]. Thus, vaccination against varicella for susceptible medical students is highly recommended.

In the UAE, vaccine against measles was introduced in 1980 and the combined vaccine against measles, mumps and rubella (MMR) in 1984–1985. The initial immunization schedule included one dose of measles vaccine at 9 months of age. In 1984, a second dose of MMR was introduced at school entry. Since 1986, the second dose of MMR vaccine was introduced at 15 months of age; the second dose at school entry was then reserved only for children who missed the dose at 15 months of age. By the end of 2004, the dose of measles vaccine at 9 months of age was omitted from the vaccination schedule; the first dose of MMR vaccine moved from 15 to 12 months of age and the second dose remained at school entry (Annual Reports, 1992, 1998, 2002, 2004, and 2010; Preventive Medicine Department, Ministry of Health, UAE). Thus, typically, Emirati students are expected to have received one dose of measles vaccine at 9 months and a second dose at 15 months. The relatively low measles seroprevalence probably reflects early vaccination against measles in the study cohort.

UAE was among the first countries to adopt the WHO EPI. Consequently, childhood vaccine-preventable diseases have been almost eliminated, with an immunization coverage reaching 98% (WHO/ Ministry of Health Annual Report, 2010). The current challenges, however, are to indentify immunization gaps in particularly susceptible groups and implement reimmunization policies to address the gaps. The data here show that 50% of students tested were susceptible to HBV and measles virus and require reimmunization. Furthermore, the results prove that the QuantiFERON®-TB test should be included in admission screening, especially in areas where BCG vaccine use is universal.

The total number of measles cases reported nationally (estimated population of about 5.4 million) between 2001 and 2010 was 539, averaging 54 cases per year. The total number of rubella cases reported nationally between 1998 and 2010 was 788, averaging 61 cases per year. The number of reported cases of mumps between 2001 and 2010, however, was much larger, averaging 342 cases per year. For 2010, the incidence rate (per 100,000) of VZV was 258.2 and 66.5 for all types of viral hepatitis.

Based on our findings, prematriculation screening tests should at least include serology for HBV and measles virus and QuantiFERON®-TB testing. Students who are nonreactive or equivocal for HBV and measles virus need reimmunization. Students with a positive or indeterminate QuantiFERON®-TB test need clinical assessment and further work-up.

## Conclusion

Significant numbers of students are susceptible to HBV and measles; therefore, pre-matriculation screening for antibodies against HBV and measles is recommended. Serological studies in preclinical years are necessary to ensure adequate protection before the students are in close contacts with patients. The endeavor is needed even in areas with reported high immunization coverage rates. Interpretation of the recently introduced QuantiFERON®-TB requires further evaluation, especially in regions of indiscriminatory use of BCG at birth. Nevertheless, all students with positive or indeterminate QuantiFERON®-TB test require thorough clinical evaluation by infectious disease consultants.

## Competing interests

The authors declare that they have no competing interests. Herewith, we confirm that there are no potential conflicts of interests and any sources of funding.

## Authors’ contributions

MSH performed study design, data collection and analysis; RH performed clinical and laboratory evaluations; ARA contributed to data analysis, literature review and manuscript writing; FAM participated in study design and manuscript writing; LA participated in study design and literature review; AKS performed data analysis and manuscript writing. All authors participated, read and approved the final manuscript.

## Pre-publication history

The pre-publication history for this paper can be accessed here:

http://www.biomedcentral.com/1471-2458/12/1047/prepub
